# Advocating for evidence in birth: Proving cause, effecting outcomes, and making the case for ‘curers’

**DOI:** 10.17157/mat.6.2.674

**Published:** 2019-09-30

**Authors:** Andrea Ford

**Keywords:** evidence, boundary object, maternity care, childbirth activism, epistemology, knowledge politics

## Abstract

The notion of ‘evidence’ circulates in two realms of current maternity care: biomedical ‘evidence-based’ obstetrics and efforts to reform conventional obstetric practices. I observed that in California's childbearing culture, ‘evidence’ is a boundary object that allows diverse actors to engage in related conversations despite fundamentally different assumptions about what evidence is or does. Sometimes these actors form productive hybrids and other times they talk past one another. This article uses recent work from the history and philosophy of science to distinguish the biomedical use of evidence, which is based on controlled experiments to prove cause and effect, from reformists' use of evidence, which foregrounds patient outcomes. Using Stengers's classification of doctors, charlatans, and curers, I discuss the role of rationality and experience in producing authoritative knowledge. Reformists' use of evidence, in effect, challenges medical power dynamics on what they perceive to be the terms of medical authority itself; in doing so, it has the potential to fundamentally alter who is the primary beneficiary of medical protocols. The challenge is continuing to use evidence in a way that doesn't simply ossify a new set of norms, but becomes increasingly capacious, flexible, specific, and patient centered.

The crowd isn’t large, maybe thirty or forty people. But energy is high. Hand-drawn rainbow prayer flags wave above us, including one I drew myself, at the BirthKeepers conference the day before. The woman speaking is standing on a platform, a sparkling turquoise cape draped around her shoulders. I had just learned the day before that turquoise is the color of the birth revolution. We are gathered outside the annual conference of the American College of Obstetricians and Gynecologists (ACOG), in San Francisco, in May of 2015.

‘We demand evidence-based care!’ she says. ‘Don’t induce for large babies, low amniotic fluid, or anything before forty-two weeks — acknowledge forty to forty-four weeks as the norm! Ask before you do something, episiotomies, putting your hands inside. This is my body, I get to decide!’ The speaker proudly proclaims that she is ‘just a mom’; she is also the founder of the Just a Mom League, which advocates for changes in maternity care from outside the medical profession. She later tells me the organization is for ‘everyone who’s willing to support moms as the primary decision maker regarding their body’.

The politics of medical decision making might seem self-evidently related to the use of evidence — good care is evidence based, while practices not based on evidence may be harmful or unnecessary, indicative of authoritative traditions rather than what is optimal for the patient. ‘Evidence-based medicine’ (often abbreviated as ‘EBM’) has become a dominant organizing paradigm for health care and medicine in the Western world over the past two decades, yet the activists in this brief vignette used evidence as a rallying point to challenge the medical community about what is optimal and who gets to decide. Evidence is not as straightforward as it seems.

In 2008, Canadian obstetrician-anthropologist Wendland published a critical medical anthropology article about evidence-based obstetrics, arguing that it undermined beneficial care for birthing women by bypassing their experience, and that of the clinician, in favor of aggregated, trial-based data that reproduced ideological biases and justified increases in cesarean section. By contrast, in my recent fieldwork on childbearing in California, ‘evidence’ was commonly marshaled to oppose obstetric conventions around medicalized birth, such as those Wendland criticizes. The idea of evidence-based medicine can be appropriated to different ends and with different relationships to the power dynamics in maternity care.

Being poorly defined yet taken for granted makes evidence both confusing and powerful. In this sense, it is a boundary object (Star and Griesemer 1989), meaning something used in different ways by different communities, and thus a site of both contestation and cooperation. Star and Griesemer (1989) define such objects as having three components: interpretive flexibility, the structure of informatic and work processes (such as maternity care systems), and the dynamic of ill-structured and more tailored uses of the objects (such as researchers specialized in evidence-based policy and ‘just moms’ casually using the term).

Star (2010, 602) later writes that boundary objects ‘are a sort of arrangement that allows different groups to work together without consensus’. Here, I highlight the potential for contestation given this lack of consensus, particularly between biomedical and reformist orientations toward obstetrics. (I use the terms ‘biomedical’ to refer to authoritative conventions of evidence-producing research within the medical profession, and ‘reformist’ to refer to those who wanted to use evidence to challenge and change conventional maternity care.) Evidence can also be a boundary object within groups, as it is among reformists, and in this context it functions more in Star’s sense of enabling cooperation.

To be clear, there is a robust discussion among medical researchers about the different kinds and qualities of evidence (see, for example, Djulbegovic and Guyatt 2017), but I am concerned with what evidence is on a more fundamental level. By examining a deeper history of evidence in science, obstetrics, and midwifery, I aim to shed light on how different actors concerned with maternity care can appear to be speaking the same language while in many ways reinforcing ideological silos. Using philosopher of science Stengers’s (2003) distinctions between doctors, charlatans, and curers, this article discusses the role of rationality and experience in producing authoritative knowledge. I claim that a significant unstated difference hinges on whether evidence points to proof of cause or patient outcomes. Is it about explanation or efficacy? The lack of clarity on this point leads to both accusatory tensions — what might be called an ‘evidence war’ — and productive alignments that have the power to positively influence childbearing. I suggest that instead of collapsing and hybridizing these legacies, explicitly recognizing evidence’s variability in order to pluralize its meanings could yield a productive epistemological diversity that further improves maternity care.

## Methods and context

This article draws on my ethnographic fieldwork on childbearing culture in California’s Bay Area (2013—2016), for which I trained and served as a doula (nonmedical birth attendant) and actively participated in classes, community events, and professional meetings aimed at doulas, midwives, nurses, doctors, childbirth educators, and childbearing people. I also conducted interviews with various people from these groups and followed articles in the popular press about maternity care. As I describe elsewhere (Ford 2017), the Bay Area’s birth culture referenced conventional divisions between obstetrics and midwifery, hospital and home birth, and ‘natural’ and ‘techno-medical’ approaches as ‘sides’ that could be at odds, yet these divisions circulated as ideas or ideals more than rigid, actual alignments. Many obstetricians, for example, appreciated the ‘midwifery model of care’ and many midwives similarly appreciated the diagnostic and emergency capacities offered by hospitals and biomedicine. Beyond ideology, practical constraints such as litigation and insurance shaped the ideals and decision-making of both practitioners and childbearing people. Conceiving of these as ‘sides’ is therefore an inaccurate simplification, but it also usefully describes how many people thought and talked about maternity care, and consequently how they related to evidence.

Being a practicing doula and anthropological researcher required juggling the practical and ideological commitments associated with each role. Since doulas tend to participate in what I call ‘reformist’ circles and efforts, I am more familiar with this perspective, though I endeavored to balance things out by, for example, regularly writing for a university medical blog and attending conferences aimed at nurses and doctors. In any case, from my feminist position as both anthropologist and doula, ‘neutrality’ was never a goal. In the Bay Area, birth reformists are a professionally heterogeneous group with varying ideological commitments and degrees of investment, and include actors as diverse as parents, doulas, midwives, obstetricians, nurses, scientists, and activists (labels which are not mutually exclusive). These reformists marshal claims about evidence-based practice to push back against hospital interventions from surgery to pharmaceuticals, arguing that existing medical practice is based on litigation, profit, and outdated social conventions, not science (see Johnson 1997; Wagner 1997).

Although ‘evidence’ is on the surface an epistemological term, reformists deploy it in a political way to contest broader power dynamics, sometimes with the tone of activist underdogs challenging a patriarchal establishment. Reformists’ dissatisfaction with the politics of medical practice and knowledge production includes critiques of litigation, liability, and the ‘pharmaco-insurance-research industrial complex’. Science studies scholar Willey (2016) describes this ‘industrial complex’ as reproducing biomedicine in a circular fashion by 1) being the main treatment people can access, and thus 2) having the best ‘evidence-based’ track record because it has been around and researched, and, therefore 3) securing the most funding for continued research and distribution. Reformists’ critiques resound with medical anthropological research that finds that medical care is often provided by large corporations and that ‘institutionalized patriarchy is a core facet of birth in the United States’, along with consumption and prioritizing safety over subjectivity (Wendland 2008, 225).

Sociologists Akrich, Leane, Roberts, and Nunes (2014) have introduced the term ‘evidence-based activism’ to describe European birth organizations’ mode of engagement, arguing that childbirth activism is characterized by ‘evidential work’ that is distinct from evidence-based policy as generally understood — that is, based on ‘objective evidence’ rooted in ‘science’. The authors invoke Latour (2008) to claim that birth activists are not producing ‘matters of fact’ but rather ‘matters of concern’, which are ‘heterogeneous assemblages of people and the objects/issues with which they are concerned’. In these assemblages, evidence ‘opens or re-opens discussion by including issues and actors that were previously excluded’ (Akrich et al. 2014, 131). These organizations attempt thereby to capture the attention of traditional stakeholders, forcing them to engage in an enlarged space of discussion. Medical anthropologists Storeng and Béhague (2014) have described this phenomenon as the rise of ‘evidence-based advocacy’ in their ethnographic account of the international Safe Motherhood Initiative. They argue that the initiative changed priorities and tactics in the past two decades, moving from rationales based on feminism and social justice to a more narrowly defined ‘numbers game’ that uses scientific evidence to bolster its authority. As a result, its policy agenda narrowed as well, focusing on practices to avert maternal deaths and quantifying outcomes in terms of benefits and costs, changes which have been met with ambivalence. In this article, I build on these convincing descriptions of the recent turn towards evidence among activists and advocates by contextualizing it in the history and philosophy of science.

There are two ways that evidence can be a boundary object, lacking consensus yet enabling cooperation. The first concerns the kind of evidence that is being used and its relative quality. In EBM this is typically illustrated as a pyramid: randomized controlled trials (RCTs) are the ‘gold standard’ of evidence in contemporary biomedical systems, with systematic reviews of RCTs at the very top (Sackett et al. 1996). Beneath those are controlled trials without randomization, followed by cohort or case-control studies, then observational studies, then opinions of respected experienced authorities, and lastly personal anecdotes. Reformists often emphasized non-RCT forms of evidence, and they tended to be less concerned with where evidence fell in this hierarchy. To be clear, I am not claiming there are any neat and necessary alliances between obstetrics and RCTs, or midwifery and observational studies; obstetrics and obstetric reform have long influenced and borrowed from each other, particularly since the professionalization of American midwifery in the 1990s. For example, there have been randomized controlled trials looking at the continuity of care in midwifery (see, for example, Forster et al. 2016), and observational studies are in the biomedical evidence pyramid.

My argument takes place on a different level that is concerned with underlying epistemological paradigms. These go unarticulated even as reformist and biomedical use of evidence blends and blurs. Evidence as a concept is naturalized as self-evident and ahistorical in both orientations. Its general association with science lends it moral and epistemological potency; among those concerned with maternity care, it was implicitly understood that no one could be ‘against evidence’. Yet significant practical and political differences stemmed from how evidence was understood. In what follows, I first describe how I saw ‘evidence’ being used by reformists to shift paradigms of knowledge and practice. Then I work closely with Stengers’s ideas to trace how contemporary understandings of evidence came into being through social processes of consolidating authority; dominant understandings consider evidence as offering proof of cause, whereas marginalized understandings point to empirical outcomes. Finally, I consider the contemporary interactions between the different concepts of evidence, referencing Stengers’s concept of ‘curers’ to consider what further ‘evidential diversity’ might look like. Given the ubiquity and centrality of evidence not only in obstetrics but in medicine generally, what ‘counts’ is a high-stakes issue, consequential for everything from everyday doctor visits to whether insurance will cover potentially life-saving treatments labeled ‘experimental’. Being more explicit about epistemological underpinnings and their genealogies can, I suggest, improve maternity care, and potentially have even broader impact.

## MANA Stats and reformist evidence

‘Midwifery is the only intervention ever proven to reduce prematurity!’ exclaimed Missy Cheyney, the medical anthropologist and home-birth midwife speaking at the 2012 Midwives Alliance of North America (MANA) conference. MANA is the most inclusive professional midwifery organization, comprising hospital-based, home-birth, certified, licensed, and traditional midwives; Cheyney was speaking about the MANA Statistics Project, which collects data about births attended by midwives, primarily occurring in homes or birth centers. Her small stature belied her larger-than-life presence, as she urged the audience of mostly midwives to enroll in the MANA Stats Project, an online birth registry that collects de-identified birth data from clients who consent to participate. MANA’s Division of Research began collecting data through an online system in 2004 and the total sample size numbered more than one hundred thousand cases by 2016 (MANA n.d.-b). This registry would enable researchers who applied for access to the data set to study and make claims about safety and risk in midwife-led births at home and in birth centers, as well as to supplement and critique the obstetric canon offered by the biomedical research industry. Existing databases do not accurately capture planned home and birth center births in most states, which also makes funding such research challenging.

This dynamic speaker enumerated many of the findings that were emerging from the studies underway and the resulting claims: more than 70 percent of women delivered not laying on their backs (squatting, hands and knees, side-lying, etc.), and 50 percent had little to no perineal damage, suggesting a possible connection between delivery position and perineal tearing. She reported the rate of cesarean section for women who went into labor intending a home or birth center birth (5 percent) and described very high rates of exclusive breastfeeding at the six-week visit (98 percent). She elaborated a careful discussion of how to represent transfers to the hospital in the statistics. In her opinion, the data showed ‘What we’re doing right’, and that ‘Midwifery works!’ She lamented the spread of dominant US models of obstetric care to other countries — ‘low resource nations are attempting to emulate our broken system’ — and mentioned partner initiatives such as the Home Birth Consensus Summit, letting audience members know where they could get a PDF pamphlet of abstracts of all known research on home birth.^1^ Her presentation of the database initiative clearly positioned it as a reformist project in which statistical evidence would be used to challenge dominant understandings of the intersection of medicine and birth, demonstrating that ‘home birth is safe’ for healthy women by comparing rates of various mortalities and morbidities with those of obstetric care.

Beginning with that MANA conference, which was the first I attended during my preliminary fieldwork, I noticed the phrase ‘evidence based’ used ubiquitously by those concerned with maternity care, including pregnant people and parents, obstetricians, researchers, midwives, doulas, childbirth educators, and community activists, both those I met in person and those whose writings I found online. In my fieldwork encounters, the ideas of evidence and evidence-based practice were used to reassure, persuade, advertise, lend legitimacy, and consolidate surety among those inclined to agree. They were used to challenge (or justify) protocols and to justify (or challenge) controversial decisions, which could potentially include all decisions in the fraught cultural landscape of childbearing! It was only later that I learned about the term’s particular usage in contemporary medicine, which led me to consider the longer history of evidence and its role in legitimating scientific knowledge. Below, I give a few illustrations to set the stage for the genealogical claims that follow.

One of the most obvious examples is the website *EvidenceBasedBirth.com,* to which I was referred frequently. For prospective parents, it offers a newsletter and free access to reports describing key studies or summarizing research on a particular topic, suggesting that parents ‘print them and bring them to your appointment to talk with your provider’ (Dekker n.d. -b). For professionals, the site runs an ‘academy’ offering training, certification, and continuing education credits. It also includes a blog and shop with branded goods. The website was founded by Rebecca Dekker, PhD, a nurse who cites her own negative birthing experience as catalyzing her realization that standard American maternity care is not based on research and, in fact, is shown by research to be harmful to healthy birthing women (Dekker n.d.-a). She aims to ‘[put] the evidence back in the hands of the people who need it the most: birthing people and their families’, instead of having it be ‘locked away in medical journals’ (Dekker n.d.-a). The reports she synthesizes often reinterpret data around hot-topic issues, such as induction and ‘overdue’ babies (Dekker 2015). Dekker describes being astounded by the success of the site, whose public traffic grew 3,500% in the first year and now supports a staff (Dekker n.d.-a).

Arguably the first evidence-focused reformist effort was the Mother Friendly Childbirth Initiative (MFCI), drafted in 1996 and updated in 2015, a consensus document MANA calls ‘ahead of its time’ (MANA n.d.-a). The MFCI has five principles — normalcy of the birth process, empowerment and autonomy [for the birthing person], doing no harm, and taking responsibility [for the practitioner] — and ten steps to implement them (Improving Birth n.d.). Regional, state, and national organizations support the initiative, notably the Coalition for Improving Maternity Services. A key facet in reformists’ use of evidence is the idea that much existing hospital birth protocol is not standardized or based on empirical evidence about patient outcomes. Many common hospital practices are critiqued for not being evidence-based — often by citing research suggesting that they are, in fact, detrimental to patient experience and outcomes — including episiotomies (cutting the perineal tissue to make way for the baby’s head), continuous fetal monitoring, routine use of pitocin (a contraction-stimulating drug), inducing labor for babies that are ‘overdue’ or ‘getting too big’, directed pushing in which the birthing person is told when to ‘bear down’, routine use of the lithotomy position (lying on one’s back), and removing newborns to the nursery.

A recent initiative aligned with the MFCI principles is the California Maternal Quality Care Collaborative (CMQCC), founded in 2006 at Stanford University School of Medicine together with the State of California in response to rising maternal mortality and morbidity rates and racial disparities (Jukelevics 2013). CMQCC has contributed to the decline of maternal mortality in California by 55 percent between 2006 and 2013, while the national maternal mortality rate has continued to rise (CMQCC n.d.-b). Its Maternal Data Center provides ‘hospitals with access to near real-time benchmarking data’ that ‘links state birth certificate data with each hospital’s patient discharge data to generate a wide range of perinatal performance metrics and quality improvement insights’, representing approximately 95 percent of all births in California (CMQCC n.d.-a). This data-driven research is quite different from a randomized controlled trial; it is epistemologically akin to record keeping, like MANA Stats, but on a much larger scale and integrated with hospital infrastructure. One of CMQCC’s founders, sociologist Morton, explained to me that one of its key initiatives was introducing emergency obstetric protocols based on outcomes data, in order to standardize what was formerly a matter of doctor or hospital convention or discretion. Some popular media credits the implementation of these protocols with California bucking the dismal national trends in maternal mortality (see Belluz 2017).

Locally, Natural Resources is a prominent brick-and-mortar resource center and store in San Francisco that offers classes, trainings, and support groups. One of its emails explains that:

When prenatal education and maternity care are not patient-centered (evidence-based research that takes into account an individual woman’s values), it is possible that the risks of labor and birth interventions may not be fully disclosed. In many hospital environments, inductions and cesarean births occur more frequently than statistically medically-necessary. The hospital prenatal courses are educating you from the hospital model of labor, birth, and perinatal care which is actually sometimes based on ‘the way it’s always been done’ rather than the most current and valid research about what supports normal and healthy labor and birth. (email dated 24 March 2016)

Natural Resources describes its own classes as ‘committed to patient-centered, evidence-based, empowered, fully-informed, and individualized education and labor/birth planning’ (email dated 24 March 2016). The collapsing together of these qualities is typical of the way I saw reformists refer to evidence, as though evidence were necessarily centered around patient experience or promoting empowerment, as opposed to an implicit doctor- or institution-centric, disempowering norm. Many critical histories and social studies point out how hospital practices evolved to suit doctors’ convenience and support a power structure in which they wield authority (see for example Davis-Floyd 1992; Ehrenreich and English 1975; Katz-Rothman 1982; Oakley 1984; Wertz and Wertz 1989). As I discuss below, biomedical descriptions of EBM as clinical practice are hardly patient centered; they mention patient preferences as something to balance against evidence, perhaps reflecting the greater role patient experience has come to play in American hospital maternity care since the 1990s, after the above-mentioned critiques were written, when EBM was invented and midwives professionalized. The correlation of evidence with patient experience, patient outcomes, clinical effectiveness, and scientific accuracy cannot be taken for granted.

Practicing as a doula during fieldwork, I frequently encountered evidence about the benefits of doula-supported birth in trainings, salons, and ‘meet the doulas’ nights for prospective clients. While these were sometimes articulated in narrative ways, like ‘doulas act as patient advocates’ or ‘doulas help partners know what to do to be supportive’, benefits were also framed evidentially, that is, in terms of statistical outcomes. The more dramatic statistics that people often cited in these circles came from a 1993 study by Klaus, Kennel, and Klaus, though a recent 2017 Cochrane review gives more moderate indicators (Bohren et al. 2017). In any case, ‘evidence’ meant rates of cesarean section, average duration of labor, percentage of women reporting dissatisfaction, or APGAR scores assessing newborn health (see Dekker 2019; Understanding Research n.d.). Referencing such evidence is part of a trend to quantify and measure ‘softer’ childbearing outcomes, such as maternal satisfaction or postpartum well-being, that were long the focus of alternative or natural birth movements and that have been getting more mainstream attention recently. For example, Harvard’s Maternal Health Task Force has initiated efforts to quantify ‘respect’ and ‘autonomy’, primarily through two survey tools: the Mothers on Respect Index and the Mother’s Autonomy in Decision-Making Scale (Hodin 2017).^2^ The task force’s blog features a post titled ‘Respect in Birth Is a Right, Not a Luxury’ that explains how maternity outcomes are better when providers are respectful, lending what might otherwise be considered a moral or ethical imperative the epistemological authority of statistically determinable outcomes (Baldwin 2016).

In some instances, even highly qualitative experiences were said to be based on evidence, illustrating the powerful appeal of evidence and its frequent invocation. Natural Resources, the San Francisco center mentioned above, advertised in an email that its infant massage class series provides ‘some sweet bonding and evidence-based research proven methods of deeper connection and attachment with your baby’. The idea that a ‘deeper connection’ could be proven to have come about stretches the boundaries of conventional usage. In a similar vein, the South Africa—based Zulu Birth Project, which reclaims African traditions around childbirth, also describes itself as evidence based. Members of this group were making a US tour and passing through the East Bay in 2014, during which they gave a talk and held two-day workshops on ‘birth work in the Zulu tradition’. Their brochure explained, ‘Our solutions are evidence based practices that return childbirth and parenting and their role in the life of the community to a state of wholeness, in deep alignment with nature and a sense of the Sacred’.^3^

A particularly vivid reference to evidence occurred at a screening of *Microbirth* (Harman and Wakeford 2014), a film depicting the microbiome’s effects on health and how it is shaped in birth and infancy, which was attended mainly by birth workers. Audience members in the discussion afterward remarked on ‘how much we don’t know’ and how it was thus a shame that ‘we interfere [excessively] instead of defaulting to nature’. A pertinent example was the practice of prescribing systemic antibiotics for birthing people with group B streptococcus (a common vaginal-rectal infection) instead of simply rinsing the birth canal with clorhexidine, an antiseptic wash equally effective at preventing the infection from spreading to the baby.

In reference to this, one attendant doula said, ‘Obviously we’re not using evidence-based practices’. When the fact was raised that there is no medical protocol for ‘vaginal swabbing’, which introduces microbes from the birth canal to a baby born by c-section, another said, ‘So let’s make one! Get doctors together, we’ll force evidence-based medicine!’ The impulse to define ‘good care’ using evidence was both pervasive and taken for granted. Evidence is both aimed at those navigating the myriad, charged decisions that confront childbearing people and marshaled as a key tactic in efforts to shape current medical practice.

Evidence can be both a rallying point and a site of contestation that gives rise to ‘evidence wars’. Exemplifying this confrontation is the popular blog *The Skeptical OB,* written by obstetrician Tuteur, which presents an exaggerated characterization of the ways reformists use evidence. She was notorious among some reformists for her often-vitriolic rhetoric and insistence on the moral corruption of ‘the natural childbirth industry’. Tuteur addresses the use of evidence in at least fifteen posts, calling the idea that obstetrics is not evidence based a ‘smear campaign’ (Tuteur 2010b); this echoes reformist accusations that professional medicine has waged a ‘smear campaign’ against midwives at various points over the past two centuries (her use of ‘industry’ likewise echoes reformist critiques of the medical industry [Tuteur 2014]). She calls reformists’ use of evidence pseudoscience (Tuteur 2012b) and a ‘double standard’: evidence is embraced when it aligns with prior ideological commitments (or profits) but dismissed when it doesn’t (Tuteur 2010a). It is hard not to think of the pot calling the kettle black, as evidence is mobilized in highly charged back-and-forth accusations, both sides reified. There are posts that point out instances in which both sides call evidence ‘insufficient’, invoking the need for providers’ expertise or the consideration of context (Tuteur 2018, 2009a), and others that show how both sides use evidence to denounce the opposition’s invocation of expertise or context (Tuteur 2013, 2009b).

Tuteur (2009b) shares my position that evidence is often a rhetorical device. In fact, she calls it a form of Orwellian ‘newspeak’, chiding the use of ‘mantras’ among reformists and claiming that ‘one of the most favored, and over used mantras is that obstetricians don’t practice evidence based medicine. Indeed, when natural childbirth advocates invoke the phrase “evidence based”, it is almost always a short hand way to criticize modern obstetrics’ (Tuteur 2012b). But Tuteur is attempting to arbitrate which ‘side’ is using evidence correctly, which is quite different from my objective of considering its rhetorical and practical function as a boundary object that can be claimed by people with different aims and assumptions, enabling people to feel like they are speaking the same language even without consensus. Evidence seems a requisite language in which to discuss decisions and preferences among those actively seeking to shape maternity care, yet its ambiguity hides the long historical conversation about who can produce authoritative evidence, and under what conditions.

## Evidence, empiricism, experience

Evidence-based medicine, often abbreviated as EBM, has been a restructuring, revitalizing, and controversial force in biomedical clinical practice since its introduction in the mid-1980s, when principles of epidemiology began to be applied to individual clinical care (Sackett et al. 1985). Generally, EBM is located at the intersection of three considerations: relevant scientific evidence, clinical judgment, and patients’ values and preferences, with two cardinal rules: not all evidence is created equal, and evidence alone is never enough (Sackett et al. 1996). *The Journal of the American Medical Association* published an oral history of EBM’s development that describes it as ushering in ‘an era of evidence’ making ‘scientific research an essential basis of medical practice’ (Chalmers et al. 2018). EBM is variously heralded as dangerously new and of ancient origin. In many ways it limits physicians’ discretion, which has been cited as both a benefit and a drawback. Medical anthropologist Lambert (2006, 2633) describes it as ‘an indeterminate and malleable range of techniques and practices characterised not by particular kinds of methodological rigour, but by the pursuit of a new approach to medical knowledge and authority’.

In their recent review article, physicians Djulbegovic and Guyatt (2017, 416) claim that ‘although EBM acknowledges a role for all empirical observations, it contends that controlled clinical observations provide more trustworthy evidence than do uncontrolled observations, biological experiments, or individual clinicians’ experiences’. Wendland (2008), the obstetrician-anthropologist mentioned above, makes a strong case for the bias inherent in the production of evidence that medical communities consider high quality and ‘objective’. Her concern with evidence-based obstetrics is that physicians are ‘basing medical decisions on evidence from randomized controlled trials and other forms of aggregate data rather than on clinical experience or expert opinion’, which bolsters a hegemonic and narrow idea of good practice because of the self-reinforcing nature of how RCTs are designed, implemented, and interpreted (Wendland 2008, 218). Wendland shows how influential obstetrics research functions to cement obstetrical authority through a rhetoric of risk, safety, and tragedy; a religious respect for technology; and a promotion of bodily mistrust. She argues that as a result, ‘technological inventions without proven benefit, such as external fetal monitoring, may be enthusiastically adopted, whereas nontechnological interventions, such as the presence of a trained support person during a woman’s labor, remain little used despite excellent evidence showing improved outcomes for mother and infant’ (Wendland 2008, 224). Her analysis of obstetricians’ use of ‘evidence’ is persuasive, yet as we have seen, reformists’ use of evidence attempts precisely to invert the tendency Wendland describes, as many invoke it to prevent doctors from making decisions based on their convenience or desire for control.

The question becomes, what counts as evidence? What are various stakeholders thinking of when they invoke it? It is productive to reframe this by asking *whose experience* counts as evidence. I have suggested elsewhere (Ford 2017) that reformist knowledge politics relies on two notions of experience: situated immediate experience (intuition) and empirical generalizable experience (evidence). ‘Experience’ has a capacious history in Western thought, serving as the foundation for modern empirical science — empiricism is the belief that valid knowledge arises from sense-experience — and filtering through ideas about religion, history, and aesthetics as well as epistemology (Jay 2005). There is certainly not space here to lay out the dynamic historical relationship between experience and authoritative knowledge production, but I will touch on three relevant points: experimentation, record keeping, and objectivity.

Historians of science Shapin and Schaffer (1985) show how scientific practice in seventeenth-century England consolidated the authority of experimental knowledge production, as opposed to rationality as a form of epistemic truth. Knowledge derived from experimentation was validated by being produced in certain authorized locations, with access restricted to ‘gentlemen’ (Shapin 1988). Only certain people could witness experiments, as the ability to give reliable testimony to experimental results was informed by classist ideas of moral superiority. For example, paid technicians, though skilled, were noncredible people akin to servants, and merchants were tainted by pecuniary interest; women, children, and ‘primitives’ were excluded by default. While experimentation did broaden scientific thought from the individual rational mind to a community of knowledge makers, it still excluded the general public who had to take experimental results on trust.

Another practice of producing and validating authoritative knowledge, also developed in England in the seventeenth century but in the realm of the ‘sciences of wealth’, is record keeping. Cultural historian Poovey (1998) shows how merchant bookkeeping has served as a metaphor for rationality since at least this period, and how the precision of arithmetic replaced the eloquence of rhetoric in creating truth and virtue. A system of carefully kept records seemed to guarantee the accuracy of what merchants recorded, and the facts produced through bookkeeping had social and epistemological effects, such as proclaiming the honesty of the merchant. This privileges the numerical/quantitative over the narrative/qualitative — but bookkeeping didn’t receive legitimacy from numbers, it helped them accrue cultural authority. Although this history helps us see why quantitative ‘facts’ seem more reliable today, bookkeeping disappeared from historical accounts of knowledge. The scientific culture developing around experimentation privileged the explanatory power of theory; record keeping is not considered knowledge production in an epistemic culture that accords prestige to abstraction (consider the difference today between mathematical abstraction and accounting’s mundane specificity).

Lastly, objectivity emerged as an ideal of knowledge production in the mid-nineteenth century. Historians of science Daston and Galison (2007) trace transformations in scientific imaging to show how the ideal of blind and passive knowledge, knowledge that bears no trace of a knower, has evolved since the mid-1800s. At this time, scientific and artistic personas became polarized. Artists express themselves, while scientists suppress themselves; earlier, these social roles were indistinct. Subjective experience became the domain of art, objective experience that of science. Objectivity became evaluated on its adherence to its own system of virtuous neutrality, which was concerned with neither broader social questions of ‘the good’ nor ‘outcomes’ as such.

Given these three facets of the ‘invention of scientific facts’ (Fleck 1935) in a general historical sense, we can turn to medicine and obstetrics in particular. Medicine only tenuously belongs to the domain of science, as it is concerned with practical outcomes that are dependent on the multifaceted and capricious human body. And within medicine, obstetrics likewise only tenuously belongs, as childbearing is not an illness and thus questionably a medical event at all. It is where medical authority is most thin. Part of the historical process of consolidating medical science as the professional authority on childbirth was distinguishing its version of empiricism from the practical ‘outcomes-based’ experience of midwives who operated without institutional training or formal protocol, and thus necessarily did much of their work by observing what practices generated which outcomes, and passing such information on to others. Literate midwives often kept records and used them to draw conclusions, engaging in a practical rationalism (see Ulrich 1991). But mere documentation of outcomes, even if meticulously recorded, became considered inadequate to constitute scientific authority. Stengers (2003) claims that authoritative knowledge in medicine, tenuous for much longer than in other branches of science, discredited not only the personal or subjective invocation of experience but also the aspirationally objective, the inadequately experimental, and the merely rational as opposed to theoretical.

Stengers (2003, 15) argues that, for modern medicine, empirical results are what charlatans produce: ‘The charlatan is henceforth defined as he who puts forward his cures as proofs’. Not all evidence is equal (as the first rule of EBM states), and only controlled experiments, ‘randomized’ to produce objectivity, are considered fully legitimate scientific proof. ‘Seeing is believing’ is not good science, nor is invoking ‘what works’. Stengers asserts that this is because causality is not proven: modern medicine has sought to prove causes in order to differentiate itself from the generations of healers who came before; successful curing alone was not enough to constitute medicine. There is a specifically modern character to this definition of the charlatan: ‘Using cures as demonstrations, he makes use of a model of scientific truth and not a tradition of the supernatural’ (Stengers 2003, 15). Charlatans aspire to scientific truth, but get it wrong.

Following Stengers, the MANA Stats project to claim legitimacy by compiling observational statistics on outcomes (record keeping, essentially) is precisely the work of contemporary charlatans, who do not belong in the experiment-oriented medical profession because they make claims about ‘what happened’ without attempting to prove why. Stengers (2003, 30) could have had MANA Stats in mind when she wrote that a modern charlatan is ‘someone who thinks that by getting results he is getting proof, conducting a “real life experiment”. … Like the doctor, this charlatan considers his activity “rational” because it is proven by the success of his treatment’. In my fieldwork, reformists seemed generally enthusiastic to explain why particular outcomes occurred, but they did not attempt to prove why scientifically, that is, experimentally. I found narrations of causality to be common, for example, that nipple stimulation releases oxytocin and oxytocin causes contractions, therefore nipple stimulation will effect the onset of labor. Such explanations are not arbitrary personal claims (‘anecdata’, as one disparaging commenter on one of Tuteur’s evidence posts phrased it), but neither are they objectively randomized quantitative proofs. They are rational, not experimental. Controlled trials, by contrast, are experiments, seeking to prove cause by eliminating all but one factor, and randomization is the closest approximation to researcher objectivity, so RCTs are the ‘gold standard’ for trustworthy experience.

Medicine has been ill-suited to applying science’s explanatory powers. Stengers (2003) describes how the body is an ambiguous and frustrating witness to science. One can be cured for the ‘wrong reasons’: the imagination, randomness, the self-limiting character of many disorders, or what became known as ‘placebo effects’. Historically, this bodily intractability — compounded by the necessary artificiality of experimental situations, the impossibility of reaching experimental equilibrium, and the messiness of variables in embodied doctor-patient interactions — made it impossible to prove causality. Medical experimentation could only disprove causes. While Stengers (2003) claims that modern medicine hopes experimentation can be redeemed — that it can eventually identify causes rather than merely eliminate them — she herself puts forth a contrary vision, claiming that medicine should dispense with the anguish over separating causes (legitimate facts) from experimental situations (mere artifacts), and should instead invent new types of facts. Suggesting we invent a medicine focused on positively identifying cures rather than merely eliminating ‘fake’ ones, Stengers acknowledges a place for experimentation, but she also insists that there must also be a place for healing, where curing should be more important than proving.

The epistemic culture of obstetrics would do well to move beyond differentiating its knowledge from ‘mere’ midwifery, as it has done historically. The hybridity and dialog between midwifery and obstetrics that has begun over the past few decades due to the efforts of reformists is, I argue, just such a process. Reformist agitation to link clinical practice and outcomes-based research does focus on curing, and it seems to me that one could generally substitute ‘outcomes based’ for ‘evidence based’ in reformist discourse. The evidence-based obstetrics Wendland (2008) decries is precisely not focused on effecting positive patient outcomes (though her analysis also includes factors like patriarchy and capitalist technology, which are to the side of the epistemological point I am making here). What Stengers (2003) calls for is what is taking shape in contemporary Bay Area maternity care, as reformists’ activism has redirected the focus of ‘good research’ to outcomes prioritizing the patient’s perspective (‘cures’ in Stengers’s terms). Obstetricians are joining midwives in seeking empirically good outcomes no matter their rationale — it is just not always palatable to the obstetric system. And reformists are making use of RCT-based research to demonstrate outcomes, and integrating EBM into midwifery education and practice.^4^ But as much as the two legacies of ‘evidence’ are influencing each other in iterative ways, they are not understood to be distinct, which, I suggest, diminishes their hybrid power.

Stengers (2003) suggests ‘at her peril’ that there should be a radical disjunction between experimenting/proving and curing/healing, so that both might be embraced without one hamstringing the possibility of the other. She warns that if proof and cure are independently valued and sought, doctors will cry out for some other way to identify charlatans, so medicine is not just arbitrary — in this case, practicable by lay midwives, citizen scientists, intuitive mothers, or a nurse with a website. Tuteur’s blog, *The Skeptical OB,* falls in line with this prediction. Such defensiveness will be especially an issue, Stengers (2003) says, if medicine loses the fiction that the suffering body ‘should’ be able to tell the difference between real medicine and fake — for example, between labor induced by pitocin and that coinciding with nipple stimulation, eating spicy food, or walking up stairs, all of which are nonmedical techniques to influence labor’s onset. Does hybridizing curing and proving hamstring both, as Stengers claims? What if, instead of hybridizing evidence, we pluralized it?

## Evidential diversity and change from within

Reformists’ appeals to evidence are claims to rationality, as opposed to superstition, intuition, magic, emotional intelligence, faith/belief, or other kinds of nonauthoritative knowing often associated with midwifery, especially historically (Davis Floyd and Sargent 1997). A rational practice of keeping records and effecting outcomes is used as a bid for inclusion, but inclusion in order to destabilize. Stengers (2003, 32) writes that rationality came into being as a way to contest and transform ‘relations of authority [and] once dominant modes of legitimation’; it still ‘does not constitute an instance of neutral consensus’ but rather ‘is an ingredient that itself changes meaning, according to whether it is aligned with the powers that maintain and reproduce the categories through which we define [the conditions of our lives], or with the social movements that interrogate and destabilise the obviousness of these categories’. If we take midwives as the figurehead for reformists, we can see that they are ‘able to invent themselves via the adjective that disqualified them’ (Stengers 2003, 33).

The invocation of rationality via evidence parallels the professionalization of American midwives in the late 1990s, during which the formulation of professional credentials, licensure, and accreditation legitimated midwives’ inclusion as medical practitioners. Since then, they have effected significant change from within the medical profession. It was this projected ability to change the establishment, and thereby improve things for the majority of women, that tipped the debate in favor of those who wanted to professionalize, against those who were concerned with whether professionalization would hamper midwives’ independence and devalue the spirit and artistry of midwifery. While American midwifery and obstetrics are not nearly as integrated as they are in many other Western countries, the inclusion of midwifery in hospital settings and its increasing professional legitimation has reshaped American maternity care. The recent hospital proliferation of family birth centers and birthing suites, expanding beyond labor and delivery wards, and of baby-friendly hospitals in which the newborn is not removed to a nursery, is at least partially in response to pressure from consumers attracted to the more ‘personal care’ a midwife offers.

This ‘personal care’ reflects an orientation around the birthing person’s perspective, which is the fundamental difference between curing and proving. What would happen if cure and care, which are necessarily centered on the patient, were integrated with RCTs and experimental practice, which are shaped by an epistemic history in which care is irrelevant to producing knowledge? Perhaps it would raise the question of whether outcomes can ever be objective or neutral. In much EBM, patients are only conceived of as having preferences that might be factored into clinicians’ decision making, not as experiencing care or being in a position to comment on its effectiveness, much less decide what the problem is that needs attention (an issue especially prominent in childbirth, which reformists categorize as nonpathological in and of itself). ‘Outcomes’ are often referenced statistically, such as lowered rates of maternal death or cesarean section, which — while certainly not against the interests of birthing people — are also not patient-centric in the more qualitative sense in which power dynamics are altered. (Recall the opening anecdote, in which the speaker elides EBM with getting to decide what happens to her body.) Shifting attention from knowledge production to outcomes could highlight the experience of childbearing people, but it doesn’t necessarily do so, and there is reason to value experimental knowledge production for its own sake. Disaggregating the two genealogies of evidence — cure and proof — could allow for more specificity in how power and knowledge interact in birth.

Lorde (1984, 112), the noted black lesbian feminist, famously argued that we cannot disrupt oppression using the logic that justifies that oppression: ‘For the master’s tools will never dismantle the master’s house. They may allow us temporarily to beat him at his own game, but they will never enable us to bring about genuine change’. By analogy, if evidence is the logic on which a racist, patriarchal medical science rests — a claim implicit in much of what my reformist interlocutors did and said — then using evidence as such only allows for the narrowest parameters of change. But evidence-as-experimental-knowledge-production differs significantly from evidence-as-patient-centered-outcomes. This illuminates a gap within the boundary object, which nonetheless still functions to mask a lack of consensus. This both explains the success of using ‘evidence’ to reform obstetrics and provides fodder for thinking about how to create deeper change.

I propose that consolidating an authoritative understanding of evidence is not in the interest of optimizing maternity care for the benefit of childbearing people. Rather, in making the concept of evidence more capacious — articulating its diverse forms, expanding the types of evidence that are considered valid, and thinking through the kinds of benefits and situations to which each is applicable — maternity care might not merely allow for these differences but draw strength from them (see Lambert 2013). Wendland (2008, 227) concludes her article on evidence-based obstetrics by calling for a feminist and situated science (Haraway 1988), one that rejects the illusion of possible objectivity: ‘Only when our vaunted scientific “objectivity” is critically reconfigured as a powerful, valuable, fallible, *and partial* contributor to medical knowledge can the imagined neutrality of medical research be confronted effectively, allowing other suppressed evidence to be brought to light’ (emphasis added). She also notes an obstacle to doing so:

in the medical literature, writing on birth at present is too easily polarized between a stubbornly naive ‘birth works’ model, on the one hand, and the fearful and technocratic ‘a chance to cut is a chance to cure’, on the other. … These discourses are at cross-purposes. Appearing in different journals, they preach only to the converted, although both are also marketed to the pregnant consumer of health care. Neither group creates a world the other recognizes as real. (Wendland 2008, 227)

While in some ways the ‘evidence wars’ I have described reproduce these silos, in other ways evidence, boundary object that it is, creates a bridge between worlds. Articulating the multiplicity within the bridge is a step towards maternity care that benefits a greater diversity of birthing people. In Lorde’s (1984, 111) words, difference is a ‘fund of necessary polarities between which our creativity can spark like a dialectic’. Such a situated feminist science is necessary in the context of abysmal American maternal outcomes and disparities, which Amnesty International (2011) calls a ‘crisis’ and which has been receiving long-overdue media attention recently (Martin et al. 2017; Villarosa 2018). This could start with separating proof from cure.

As a next step, what if evidentiary practice were expanded to include the nonrational? Stengers (2003, 30) also has a category into which midwives who do not seek belonging via rationality might fall, a third category of ‘curers’ who ‘are not haunted by the idea of being able to disqualify others, but rather who have cultivated an “influencing practice”’. Such curers are not concerned with being rational (as a charlatan is), much less with proving (as a doctor-scientist is); Stengers asks if modern medicine does not indeed have something to learn from them. One of the older midwives I spoke with during fieldwork, who was a pillar of the local birth community and the natural birth movement in the 1970s, explained to me that ‘pre-stats’ she and her cohort just had a feeling that home birth was ok; they didn’t feel the need to prove it nor to consolidate a best practice, as ‘the nature of midwifery appeals to independent minds, and there will be diverse opinions. … We practice from our own innate wisdom, not protocols’. Such wisdom is a type of influencing expertise. Stengers (2003) writes that while anyone can use suggestion, imagination, or placebo effects, influence is something only experts can do.

Interestingly enough, some EBM assertions of the importance of clinician expertise sound similar, as they advocate for the role of ‘intuitive and experiential thinking, which characterize expert judgment’ (Djulbegovic and Guyatt 2017, 420). The phrase ‘evidence informed’ is being posed as an alternative to ‘evidence based’ across fields. A professional article asserts that the nonrational has a place in sensible midwifery alongside the rational (Parratt and Fahy 2008). Speaking at a 2016 Bay Area Doula Project meeting, pioneering East Bay midwife Peggy Vincent highlighted her detailed record keeping, rejected the label ‘scientist’, and called for evidence-based practice, saying, perhaps too simplistically, that ‘Midwifery is an art; obstetrics is a science. We lost sight of the art, and that’s the problem’. The distinction needn’t be so stark; there is, after all, a history of physician practice as an art form, as well (Solomon 2015). Making evidence more capacious and specific would not only make room for artistry but also allow social justice and feminism to have authoritative roles in determining best practices.

## Footnotes

1 Available at https://mana.org/research/homebirth-safety, accessed 25 July 2019.

2 See also the Birth Place Research Quality Index, by the University of British Columbia’s Birth Place Lab, which is designed to help families, doctors, midwives, and policymakers interpret research in a systematic way in order to evaluate the safety of birth place options: http://www.birthplacelab.org/tools/, accessed 21 August 2018.

3 Copied from now-defunct website http://www.zulubirthproject.com/ on 15 September 2014. The Zulu birth project ran from 2014—2017. An archived version of this webpage (from 13 January 2016) is availablen via the Internet Archive at: https://web.archive.org/web/20160819024835/http://www.zulubirthproject.com/zulubirthproject/hello-world/.

4 See, for example, Anderson and Stone (2012); Spilby and Munroe (2009). One of the earliest efforts in this direction was the 1989 book A Guide to Effective Care in Pregnancy and Childbirth, which reviewed three thousand RCTs to identify effective practices and improve the quality of maternity care (as cited in Johnson 1997).
